# Bis(benzyl­sulfan­yl)methane

**DOI:** 10.1107/S1600536810019641

**Published:** 2010-05-29

**Authors:** Hojin Yang, Tae Ho Kim, Suk-Hee Moon, Jineun Kim

**Affiliations:** a Department of Chemistry and Research Institute of Natural Sciences, Gyeongsang National University, Jinju 660-701, Republic of Korea; b Subdivision of Food Science, Kyungnam College of Information and Technology, Busan 616-701, Republic of Korea

## Abstract

In the title compound, C_15_H_16_S_2_, the structure of the dithioalkyl chain is a helix with an all-*cis* conformation. The dihedral angle between the mean planes of the terminal aromatic rings is 74.60 (4)°. In the crystal structure, weak C—H⋯π inter­actions contribute to the stabilization of the packing.

## Related literature

For the synthesis of the title ligand, see: Cohen *et al.* (1980 ▶). For related structures, see: Li *et al.* (2005 ▶); Tanaka & Ajiki (2005 ▶).
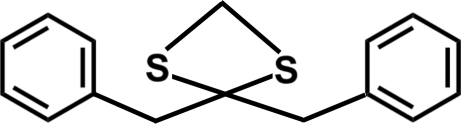


## Experimental

### 

#### Crystal data

C_15_H_16_S_2_
*M* *_r_* = 260.40
Monoclinic, 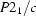

*a* = 5.5146 (1) Å
*b* = 12.2628 (3) Å
*c* = 20.0128 (5) Å
β = 101.156 (1)°
*V* = 1327.78 (5) Å^3^
*Z* = 4
Mo *K*α radiation
μ = 0.38 mm^−1^
*T* = 173 K
0.22 × 0.15 × 0.15 mm

#### Data collection

Bruker APEXII CCD diffractometer
Absorption correction: multi-scan (*SADABS*; Sheldrick, 1996 ▶) *T*_min_ = 0.922, *T*_max_ = 0.946
12942 measured reflections
3335 independent reflections
2977 reflections with *I* > 2σ(*I*)
*R*_int_ = 0.033

#### Refinement

*R*[*F* ^2^ > 2σ(*F* ^2^)] = 0.031
*wR*(*F* ^2^) = 0.082
*S* = 1.04
3335 reflections
155 parameters
H-atom parameters constrained
Δρ_max_ = 0.30 e Å^−3^
Δρ_min_ = −0.29 e Å^−3^

### 

Data collection: *APEX2* (Bruker, 2006 ▶); cell refinement: *SAINT* (Bruker, 2006 ▶); data reduction: *SAINT*; program(s) used to solve structure: *SHELXTL* (Sheldrick, 2008 ▶); program(s) used to refine structure: *SHELXTL*; molecular graphics: *SHELXTL* and *DIAMOND* (Brandenburg, 1998 ▶); software used to prepare material for publication: *SHELXTL*.

## Supplementary Material

Crystal structure: contains datablocks global, I. DOI: 10.1107/S1600536810019641/sj5008sup1.cif

Structure factors: contains datablocks I. DOI: 10.1107/S1600536810019641/sj5008Isup2.hkl

Additional supplementary materials:  crystallographic information; 3D view; checkCIF report

## Figures and Tables

**Table 1:** Hydrogen-bond geometry (Å, °) *Cg* is the centroid of the C1–C6 ring.

*D*—H⋯*A*	*D*—H	H⋯*A*	*D*⋯*A*	*D*—H⋯*A*
C13—H13⋯*Cg*^i^	0.95	2.85	3.71	151

Symmetry code: (i) 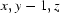.

**Fig. 1.**
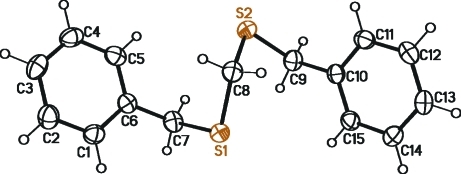
The molecular structure of the title compound, showing displacement ellipsoids drawn at the 50% probability level.

**Fig. 2.**
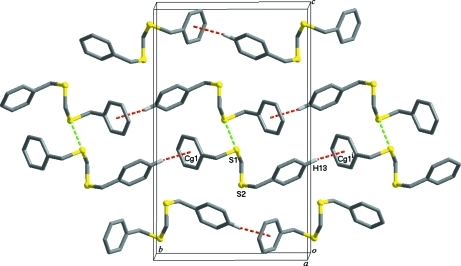
Intermolecular C—H···π (red dotted lines) and S···S (green dotted lines) interactions in the title compound. All H atoms except those related to intermolecular interactions have been omitted for clarity. Cg is the centroid of the C1/C2/C3/C4/C5/C6 ring. [Symmetry codes: (i) x, -1+y, z]

## supplementary crystallographic information

### Comment

Dithio acetals (RSCH_2_SR) have received considerable attention in the literature (Li *et al.*,  2005; Tanaka & Ajiki, 2005). We report herein the crystal structure of the title compound. In asymmetric unit, the conformation of dithioalkyl chain is all *cis* and the dihedral angle between the aromatic rings is 74.60 (4)°. In the crystal structure (Fig. 1), the bond lengths and angles are within normal ranges.

A weak C13—H13···Cg = 2.85 Å interaction (Cg is the centroid of the C1···C6 ring) is observed, Table 1. Weak intermolecular S···S interactions with 3.4732 (6)Å also exist. These intermolecular interactions may be effective in the stabilization of the structure, Fig. 2.

### Experimental

The title compound was synthesised according to the published procedure (Cohen *et al.*, 1980) and recrystallized from petroleum ether.

### Refinement

All H-atoms were positioned geometrically and refined using a riding model with d(C—H) = 0.95 Å, *U*_iso_ =1.2*U*_eq_(C) for aromatic and 0.99 Å, *U*_iso_ = 1.2*U*_eq_(C) for the CH_2_ atoms.

### Crystal data

**Table:**

C_15_H_16_S_2_	*F*(000) = 552
*M**_r_* = 260.40	*D*_x_ = 1.303 Mg m^−^^3^
Monoclinic, *P*2_1_/*c*	Mo *K*α radiation, λ = 0.71073 Å
Hall symbol: -P 2ybc	Cell parameters from 7825 reflections
*a* = 5.5146 (1) Å	θ = 2.7–28.4°
*b* = 12.2628 (3) Å	µ = 0.38 mm^−^^1^
*c* = 20.0128 (5) Å	*T* = 173 K
β = 101.156 (1)°	Block, colourless
*V* = 1327.78 (5) Å^3^	0.22 × 0.15 × 0.15 mm
*Z* = 4	

### Data collection

**Table:**

Bruker APEXII CCD diffractometer	3335 independent reflections
Radiation source: fine-focus sealed tube	2977 reflections with *I* > 2σ(*I*)
graphite	*R*_int_ = 0.033
Detector resolution: 10.0 pixels mm^-1^	θ_max_ = 28.4°, θ_min_ = 2.0°
φ and ω scans	*h* = −7→7
Absorption correction: multi-scan (*SADABS*; Sheldrick, 1996)	*k* = −12→16
*T*_min_ = 0.922, *T*_max_ = 0.946	*l* = −24→26
12942 measured reflections	

### Refinement

**Table:**

Refinement on *F*^2^	Secondary atom site location: difference Fourier map
Least-squares matrix: full	Hydrogen site location: inferred from neighbouring sites
*R*[*F*^2^ > 2σ(*F*^2^)] = 0.031	H-atom parameters constrained
*wR*(*F*^2^) = 0.082	*w* = 1/[σ^2^(*F*_o_^2^) + (0.0369*P*)^2^ + 0.4363*P*] where *P* = (*F*_o_^2^ + 2*F*_c_^2^)/3
*S* = 1.04	(Δ/σ)_max_ < 0.001
3335 reflections	Δρ_max_ = 0.30 e Å^−^^3^
155 parameters	Δρ_min_ = −0.29 e Å^−^^3^
0 restraints	Extinction correction: *SHELXTL* (Sheldrick, 2008), Fc^*^=kFc[1+0.001xFc^2^λ^3^/sin(2θ)]^-1/4^
Primary atom site location: structure-invariant direct methods	Extinction coefficient: 0.030 (2)

### Special details

**Table:**

Geometry. All esds (except the esd in the dihedral angle between two l.s. planes) are estimated using the full covariance matrix. The cell esds are taken into account individually in the estimation of esds in distances, angles and torsion angles; correlations between esds in cell parameters are only used when they are defined by crystal symmetry. An approximate (isotropic) treatment of cell esds is used for estimating esds involving l.s. planes.
Refinement. Refinement of *F*^2^ against ALL reflections. The weighted *R*-factor wR and goodness of fit *S* are based on *F*^2^, conventional *R*-factors *R* are based on *F*, with *F* set to zero for negative *F*^2^. The threshold expression of *F*^2^ > σ(*F*^2^) is used only for calculating *R*-factors(gt) etc. and is not relevant to the choice of reflections for refinement. *R*-factors based on *F*^2^ are statistically about twice as large as those based on *F*, and *R*- factors based on ALL data will be even larger.

### Fractional atomic coordinates and isotropic or equivalent isotropic displacement parameters (Å^2^)

**Table:**

	*x*	*y*	*z*	*U*_iso_*/*U*_eq_	
S1	0.63212 (6)	0.46872 (3)	0.430321 (16)	0.03123 (10)	
S2	0.80471 (6)	0.43167 (3)	0.294901 (16)	0.03327 (11)	
C1	0.7958 (2)	0.73297 (11)	0.47700 (6)	0.0318 (3)	
H1	0.6990	0.7130	0.5095	0.038*	
C2	0.9857 (3)	0.80750 (11)	0.49456 (7)	0.0369 (3)	
H2	1.0197	0.8377	0.5391	0.044*	
C3	1.1262 (3)	0.83815 (11)	0.44745 (8)	0.0376 (3)	
H3	1.2579	0.8887	0.4597	0.045*	
C4	1.0740 (3)	0.79496 (11)	0.38253 (7)	0.0371 (3)	
H4	1.1678	0.8170	0.3498	0.045*	
C5	0.8848 (2)	0.71936 (11)	0.36495 (6)	0.0324 (3)	
H5	0.8508	0.6897	0.3203	0.039*	
C6	0.7447 (2)	0.68671 (10)	0.41215 (6)	0.0269 (2)	
C7	0.5451 (2)	0.60237 (11)	0.39470 (7)	0.0319 (3)	
H7A	0.4995	0.5959	0.3445	0.038*	
H7B	0.3970	0.6276	0.4114	0.038*	
C8	0.8761 (2)	0.43180 (11)	0.38662 (7)	0.0303 (3)	
H8A	0.9344	0.3579	0.4019	0.036*	
H8B	1.0158	0.4826	0.4014	0.036*	
C9	0.5351 (2)	0.34398 (11)	0.27868 (6)	0.0320 (3)	
H9A	0.3992	0.3808	0.2956	0.038*	
H9B	0.4822	0.3351	0.2288	0.038*	
C10	0.5717 (2)	0.23235 (10)	0.31064 (6)	0.0263 (2)	
C11	0.7532 (2)	0.16136 (11)	0.29690 (6)	0.0309 (3)	
H11	0.8598	0.1839	0.2675	0.037*	
C12	0.7799 (2)	0.05829 (11)	0.32574 (7)	0.0327 (3)	
H12	0.9041	0.0106	0.3158	0.039*	
C13	0.6268 (2)	0.02439 (11)	0.36880 (7)	0.0324 (3)	
H13	0.6452	−0.0463	0.3885	0.039*	
C14	0.4464 (3)	0.09429 (11)	0.38292 (7)	0.0344 (3)	
H14	0.3406	0.0716	0.4125	0.041*	
C15	0.4196 (2)	0.19762 (11)	0.35389 (7)	0.0307 (3)	
H15	0.2951	0.2451	0.3639	0.037*	

### Atomic displacement parameters (Å^2^)

**Table:**

	*U*^11^	*U*^22^	*U*^33^	*U*^12^	*U*^13^	*U*^23^
S1	0.03604 (18)	0.02830 (18)	0.03133 (17)	−0.00231 (12)	0.01147 (13)	−0.00010 (12)
S2	0.04070 (19)	0.02943 (18)	0.03283 (17)	−0.00462 (13)	0.01497 (13)	0.00064 (12)
C1	0.0353 (6)	0.0333 (7)	0.0270 (6)	−0.0013 (5)	0.0064 (5)	0.0029 (5)
C2	0.0422 (7)	0.0336 (7)	0.0322 (6)	−0.0033 (6)	0.0008 (5)	0.0007 (5)
C3	0.0336 (7)	0.0275 (7)	0.0505 (8)	−0.0018 (5)	0.0048 (6)	0.0079 (6)
C4	0.0395 (7)	0.0316 (7)	0.0445 (7)	0.0056 (6)	0.0185 (6)	0.0113 (6)
C5	0.0390 (7)	0.0293 (6)	0.0299 (6)	0.0089 (5)	0.0093 (5)	0.0039 (5)
C6	0.0270 (6)	0.0240 (6)	0.0285 (5)	0.0060 (5)	0.0026 (4)	0.0028 (4)
C7	0.0269 (6)	0.0302 (6)	0.0368 (6)	0.0046 (5)	0.0018 (5)	−0.0015 (5)
C8	0.0263 (6)	0.0291 (6)	0.0349 (6)	0.0019 (5)	0.0042 (5)	0.0005 (5)
C9	0.0345 (6)	0.0295 (6)	0.0302 (6)	−0.0001 (5)	0.0019 (5)	0.0020 (5)
C10	0.0264 (5)	0.0257 (6)	0.0251 (5)	−0.0019 (5)	0.0009 (4)	−0.0029 (4)
C11	0.0309 (6)	0.0340 (7)	0.0288 (6)	−0.0006 (5)	0.0085 (5)	−0.0032 (5)
C12	0.0320 (6)	0.0297 (6)	0.0359 (6)	0.0043 (5)	0.0051 (5)	−0.0072 (5)
C13	0.0355 (6)	0.0231 (6)	0.0364 (6)	−0.0019 (5)	0.0011 (5)	−0.0015 (5)
C14	0.0347 (7)	0.0305 (7)	0.0399 (7)	−0.0041 (5)	0.0120 (5)	0.0012 (5)
C15	0.0277 (6)	0.0281 (6)	0.0374 (6)	0.0011 (5)	0.0087 (5)	−0.0027 (5)

### Geometric parameters (Å, °)

**Table:**

S1—C8	1.7988 (13)	C7—H7B	0.9900
S1—C7	1.8144 (14)	C8—H8A	0.9900
S2—C8	1.8013 (13)	C8—H8B	0.9900
S2—C9	1.8125 (14)	C9—C10	1.5079 (17)
C1—C2	1.3831 (19)	C9—H9A	0.9900
C1—C6	1.3942 (17)	C9—H9B	0.9900
C1—H1	0.9500	C10—C15	1.3841 (18)
C2—C3	1.383 (2)	C10—C11	1.3934 (18)
C2—H2	0.9500	C11—C12	1.3854 (19)
C3—C4	1.381 (2)	C11—H11	0.9500
C3—H3	0.9500	C12—C13	1.382 (2)
C4—C5	1.389 (2)	C12—H12	0.9500
C4—H4	0.9500	C13—C14	1.383 (2)
C5—C6	1.3898 (18)	C13—H13	0.9500
C5—H5	0.9500	C14—C15	1.3899 (19)
C6—C7	1.5016 (18)	C14—H14	0.9500
C7—H7A	0.9900	C15—H15	0.9500
			
C8—S1—C7	101.67 (6)	S2—C8—H8A	108.0
C8—S2—C9	101.13 (6)	S1—C8—H8B	108.0
C2—C1—C6	120.80 (12)	S2—C8—H8B	108.0
C2—C1—H1	119.6	H8A—C8—H8B	107.2
C6—C1—H1	119.6	C10—C9—S2	115.14 (9)
C1—C2—C3	120.19 (13)	C10—C9—H9A	108.5
C1—C2—H2	119.9	S2—C9—H9A	108.5
C3—C2—H2	119.9	C10—C9—H9B	108.5
C4—C3—C2	119.65 (13)	S2—C9—H9B	108.5
C4—C3—H3	120.2	H9A—C9—H9B	107.5
C2—C3—H3	120.2	C15—C10—C11	118.46 (12)
C3—C4—C5	120.26 (13)	C15—C10—C9	119.80 (11)
C3—C4—H4	119.9	C11—C10—C9	121.73 (11)
C5—C4—H4	119.9	C12—C11—C10	120.68 (12)
C4—C5—C6	120.61 (12)	C12—C11—H11	119.7
C4—C5—H5	119.7	C10—C11—H11	119.7
C6—C5—H5	119.7	C13—C12—C11	120.38 (12)
C5—C6—C1	118.46 (12)	C13—C12—H12	119.8
C5—C6—C7	121.28 (11)	C11—C12—H12	119.8
C1—C6—C7	120.25 (12)	C12—C13—C14	119.43 (12)
C6—C7—S1	113.86 (8)	C12—C13—H13	120.3
C6—C7—H7A	108.8	C14—C13—H13	120.3
S1—C7—H7A	108.8	C13—C14—C15	120.14 (13)
C6—C7—H7B	108.8	C13—C14—H14	119.9
S1—C7—H7B	108.8	C15—C14—H14	119.9
H7A—C7—H7B	107.7	C10—C15—C14	120.91 (12)
S1—C8—S2	117.33 (7)	C10—C15—H15	119.5
S1—C8—H8A	108.0	C14—C15—H15	119.5
			
C6—C1—C2—C3	0.7 (2)	C9—S2—C8—S1	−53.62 (9)
C1—C2—C3—C4	0.8 (2)	C8—S2—C9—C10	−55.87 (11)
C2—C3—C4—C5	−1.4 (2)	S2—C9—C10—C15	123.62 (11)
C3—C4—C5—C6	0.4 (2)	S2—C9—C10—C11	−57.57 (14)
C4—C5—C6—C1	1.09 (18)	C15—C10—C11—C12	0.22 (18)
C4—C5—C6—C7	−177.97 (12)	C9—C10—C11—C12	−178.61 (11)
C2—C1—C6—C5	−1.67 (19)	C10—C11—C12—C13	−0.15 (19)
C2—C1—C6—C7	177.40 (12)	C11—C12—C13—C14	0.00 (19)
C5—C6—C7—S1	103.14 (12)	C12—C13—C14—C15	0.1 (2)
C1—C6—C7—S1	−75.91 (14)	C11—C10—C15—C14	−0.12 (18)
C8—S1—C7—C6	−64.96 (10)	C9—C10—C15—C14	178.72 (12)
C7—S1—C8—S2	−56.40 (9)	C13—C14—C15—C10	0.0 (2)

### Hydrogen-bond geometry (Å, °)

**Table:**

Cg is the centroid of the C1–C6 ring.

**Table:**

*D*—H···*A*	*D*—H	H···*A*	*D*···*A*	*D*—H···*A*
C13—H13···Cg^i^	0.95	2.85	3.71	151

Symmetry codes: (i) *x*, *y*−1, *z*.
